# Quantum Confined Stark Effect on the Linear and Nonlinear Optical Properties of SiGe/Si Semi Oblate and Prolate Quantum Dots Grown in Si Wetting Layer

**DOI:** 10.3390/nano11061513

**Published:** 2021-06-08

**Authors:** Varsha, Mohamed Kria, Jawad El Hamdaoui, Laura M. Pérez, Vinod Prasad, Mohamed El-Yadri, David Laroze, El Mustapha Feddi

**Affiliations:** 1 Department of Physics and Astrophysics, University of Delhi, Delhi-110007, India; varsha.yadav610@gmail.com; 2 Department of Physics, Kalindi College, University of Delhi, Delhi-110008, India; 3 Group of Optoelectronic of Semiconductors and Nanomaterials, ENSAM, Mohammed V University in Rabat, Rabat 10100, Morocco; mohamedkria1@gmail.com (M.K.); jawadelhamdaoui11@gmail.com (J.E.H.); md.yadri@gmail.com (M.E.-Y.); 4 Instituto de Alta Investigación, CEDENNA, Universidad de Tarapacá, Casilla 7 D, Arica 1000000, Chile; dlarozen@uta.cl; 5 Department of Physics, Swami Shraddhanand College, University of Delhi, Delhi-110036, India

**Keywords:** prolate quantum dot (QD), oblate quantum dot (QD), absorption coefficient (AC), second harmonic generation (SHG), electric field

## Abstract

We have studied the parallel and perpendicular electric field effects on the system of SiGe prolate and oblate quantum dots numerically, taking into account the wetting layer and quantum dot size effects. Using the effective-mass approximation in the two bands model, we computationally calculated the extensive variation of dipole matrix (DM) elements, bandgap and non-linear optical properties, including absorption coefficients, refractive index changes, second harmonic generation and third harmonic generation as a function of the electric field, wetting layer size and the size of the quantum dot. The redshift is observed for the non-linear optical properties with the increasing electric field and an increase in wetting layer thickness. The sensitivity to the electric field toward the shape of the quantum dot is also observed. This study is resourceful for all the researchers as it provides a pragmatic model by considering oblate and prolate shaped quantum dots by explaining the optical and electronic properties precisely, as a consequence of the confined stark shift and wetting layer.

## 1. Introduction

The science of zero-dimensional semiconductor nanomaterial structures, quantum dots (QDs), has revolutionized the research of fabrication of optoelectronic devices as they have exquisite optical and electronic properties [1,2,3]. The renovation of the fabrication capabilities opened the arena of different shapes and sizes of QDs that lead to the vicissitude in the electronic and nonlinear optical properties. Many theorists and experimentalists have undertaken intensive work to achieve innovation in this domain, also called bandgap engineering [4,5,6,7,8,9,10]. Experimentally, during epitaxial growth, QDs arise at the wetting layer (WL) of the material. However, during the growth process, the constraints generated by lattice mismatch of different materials and the strain of WL cause non-symmetrical and non-homogenous shapes. Thus, different shapes with different size dispersions can be observed by TEM imaging: hemispherical, disk, cylinder, lens, ring, conical, pyramidal, dome, oblate and prolate or semi oblate and prolate [11,12,13,14,15,16,17,18,19,20].

The optoelectronic properties of these different shapes attracted much interest and have been the subject of intensive theoretical investigations. Some authors have used orthogonal curvilinear coordinates to solve exactly the Schrdinger equation of QDs with elliptical symmetry. The elliptic coordinates have been used to solve the single-particle problem in an elliptical dot [21]. Two interesting papers of Cantele et al. [9,22] constitute important references in such systems; they have used the spheroidal coordinates to solve the Schrdinger equation of the single–particle problem in oblate and prolate spheroidal QDs. They have shown the strong influence of the electron–electron correlation term on the QD anisotropy with the study of the one-and two-electron ground states in ellipsoidal QDs. By using the parabolic coordinates, Even and Loualiche [23] have found analytical expressions of energy levels and the wave functions of one particle in a lens-shaped QD. The energy levels of a donor impurity have been determined in the case of a parabolic QD [24] by using parabolic coordinates. Assaid et al. have determined variationally the energy levels of a donor impurity in the symmetrical paraboloidal QD. More recently, by using the effective mass and parabolic band approximations, they have determined analytically the energies of the fundamental and few low lying states of a single electron confined in a paraboloidal quantum lens [25]. They further studied the Stark effect and the polarizability of shallow-donor impurity located in the center of a lens-shaped QD by a variational method [26]. Many other properties for different shapes have been analyzed. For instance, the nonlinear optical properties of a two dimensional elliptical QD were investigated by Rezaei et al. [27], with the use of the compact-density matrix formalism and an iterative method. The authors confirmed that, along with the intensity of light, the geometrical shape and size greatly influence the optical absorption coefficient (AC) and refractive index changes (RIC) of the system. In Reference [28], with the adiabatic approximation, the electron states and light absorption are investigated for the study of strongly oblate and strongly prolate ellipsoidal QDs in the presence of electrical and magnetic fields. Shi and Yan [29] studied the system of an exciton bound to an ionized donor impurity in GaAs ellipsoidal QDs under shape and electric field effects within the effective-mass approximation using a variational method in the framework of perturbation theory. Dujardin et al. [30], using the variational method, studied the excitonic binding energy in prolate and oblate spheroidal QDs. The authors found that the binding of the spherical case is a minimum and it increases when the deformation is accentuated, and explained that the bandgap is also tuned with the help of the shape of the QD.

The electric field effect on QD semiconductors continues to attract much interest [26,31,32,33,34,35,36,37,38,39]. It leads to the well-known effect, the quantum-confined Stark effect (QCSE), characterized by a red-shift many times greater than the electron-hole binding energy. Generally speaking, the investigations related to the effect of the electric field on confined carriers show that two conflicting behaviors can exist: the redshift of excitonic absorption, because under an electric field, band bending and tilted band structure are induced, which also leads to a dropped electron energy level and the hole sub-band energy level increases, which induces a redshift. The other effect is the polarization of the exciton. The electron and hole push back due to the electrostatic forces, which diminishes the Coulomb attraction and gives rise to the electron-hole pair energy which corresponds to a blueshift [40]. This is why the world market for nonlinear optical crystals is experiencing great expansion. A large community of researchers carried out an intensive study on the effect of the electric field on the optical properties of the system [41,42,43,44,45,46,47]. The analysis of optical behavior is crucial as it gives an insight into the intersubband transition in a controlled manner. The optical AC in a ring-shaped elliptical QD with the effects of hydrogenic impurity, electric and magnetic fields is presented in Reference [48]. Using the finite element method, they found that with the increasing electric field, the optical absorption increases for impurity situated at the center.

It is very important to underline that a simple overview of the literature shows that, in the case of the process of growth of SiGe, the most probable shapes obtained are semi oblate or semi prolate. They are considered the most realistic models that are close to the experimental samples. The semiconducting material $Si_{1-\eta }Ge_{\eta }$, (where η is the concentration of Ge) has been used for many electronic devices due to its large density, high dielectric constant and excellent optical properties [49,50,51,52,53]. Their nonlinear optical properties like AC, RIC, second harmonic generation (SHG) and third harmonic generation (THG) provide a thorough insight into the mechanism of the optical response. Among the many interesting results, we cite Carletti et al. [54], who have studied the nonlinear optical properties of SiGe waveguides in the mid-infrared. Duque et al. [55] have determined the Intersubband linear and nonlinear optical response of the delta-doped SiGe quantum well. Lacava et al. [56] demonstrated the nonlinear silicon and germanium photonic signal processing devices for future optical networks. Femtosecond time-resolved pump-probe spectroscopy is used to investigate the ultrafast carrier dynamics of Ge/SiGe quantum wells grown on a Si substrate by Lange et al. [57]. Soref et al. investigated the electro-optical and nonlinear optical coefficients of an ordered group IV semiconductor alloys [58].

To our knowledge, there has been no study concerning the effect of the electric field on the nonlinear optical properties of the SiGe/Si with these realistic shapes. Recently, we have obtained the nonlinear optical properties of SiGe/Si for oblate and prolate QD for the first time [20]. In this study, we have investigated the effect of the electric field and WL in prolate and oblate QDs for $Si_{1-\eta }Ge_{\eta }$ material for η=0.3 for the WL surrounded by Si matrix with varying size. We have found an interesting behavioral difference in prolate and oblate QD for nonlinear optical properties, especially in SHG and THG. We found a strong dependence of nonlinear optical properties on the electric field, WL, the geometrical size of QD, and also the shape of the QD. The paper has a detailed theory and model of the system with a complete formulation and detailed results. We have concluded our results in the final section of the conclusion.

## 2. Theory and Model

**Figure 1:**
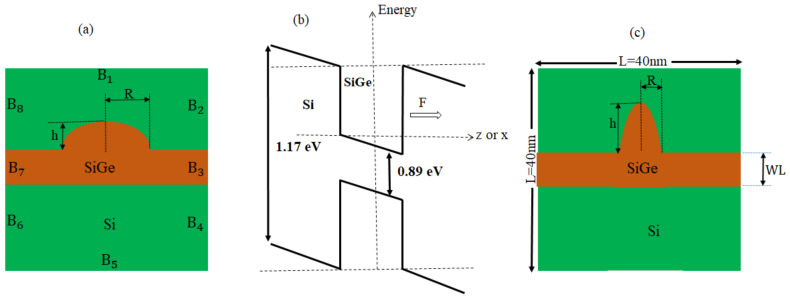
Cross section of oblate QD (**a**), Energy band diagram of QD (**b**), and prolate QD (**c**).

Let us consider $Si_{0.7}Ge_{0.3}/Si$ semi oblate and semi prolate QDs surrounded by an Si matrix, deposited on thick SiGe WL, see Figure 1. The system is subjected to a steady electric field (F), which can be taken in parallel (along x-axis) $\vec{F}=\left(F,0,0\right)$ or perpendicular (along z-axis) $\vec{F}=\left(0,0,F\right)$.

The eigen-energies and corresponding wave functions are a solution of the Schrödinger equation:

(1) $$\frac{-\hbar^{2}}{2}\nabla \left[\frac{1}{m_{i}^{*}\left(r\right)}\nabla \psi \left(r\right)\right]+V_{w}^{e}\left(r\right)\psi \left(r\right)-W\psi \left(r\right)=E\psi \left(r\right),$$

where ψ(r) and *E* are the wave functions and the energy levels respectively. $m_{i}^{*}$ is the position dependent effective mass of the electron, that is, different for the matrix and QD region given as:

(2) $$m_{i}^{*}=\left\{\begin{array}{cc} m_{SiGe}^{*} & \;\;\;\;\;\mathrm{inside}\;\mathrm{the}\;\mathrm{core}\;\mathrm{and}\;\mathrm{the}\;\mathrm{WL}\\ m_{Si}^{*} & \;\;\mathrm{otherwise}. \end{array}\right.$$

Similarly, the confining potential energy, $V_{w}^{e}\left(X,Y,Z\right)$ is given by:

(3) $$V_{w}^{e}\left(X,Y,Z\right)=\left\{\begin{array}{cc} 0 & \;\;\;\;\;\mathrm{inside}\;\mathrm{the}\;\mathrm{core}\;\mathrm{and}\;\mathrm{the}\;\mathrm{WL}\\ E_{g}\left(Si\right)-E_{g}\left(Si_{1-\eta }Ge_{\eta }\right) & \;\;\mathrm{otherwise}, \end{array}\right.$$

where $E_{g}\left(Si\right)=1.17$ eV. The dependence on the material composition η and temperature is introduced by [44]:

(4) $$E_{g}^{SiGe}=E_{g}^{Si}.\left(1-\eta \right)+E_{g}^{Ge}.\eta +C_{g}.\left(1-\eta \right)\eta -\frac{\alpha .T^{2}}{\beta +T}$$

where $C_{g}=-0.4$ eV, $\alpha =4.73\times 10^{-4}$ eV/K and β=636 K.

For η<0.85, the $Si_{1-\eta }Ge_{\eta }$ alloys are considered to be Si-like material: The surfaces of equal energy are ellipsoid, the effective mass of electron $m_{SiGe}^{*}=0.26m_{0}$ [59].

The dipolar energy operator $W=e\vec{F}.\vec{r}$, where *e* is the absolute value of the elementary charge of electron. In our case it can take the two expressions according to the orientation of the electric field

(5) $$W=\left\{\begin{array}{cc} -eFx & \;\;\mathrm{when}\;F\;\mathrm{is}\;\mathrm{parallel}\;\mathrm{to}\;\mathrm{the}\;\mathrm{WL}\\ -eFz & \;\;\mathrm{when}\;F\;\mathrm{is}\;\mathrm{perpendicular}\;\mathrm{to}\;\mathrm{the}\;\mathrm{WL} \end{array}\right.$$

The analytical solution of the Schrödinger equation for these geometrical shapes is arduous. We have used the finite element method with the proper choice of boundary conditions as per the physical condition to study our system as shown in Figure 1.

In order to analyze the optical properties, we use the well known formalism based on the density matrix approach [60,61,62] which allows us to determine, the linear and non-linear (third-order) ACs and RICs. The total AC is given as:

(6) $$\alpha \left(\omega ,I\right)=\alpha^{\left(1\right)}\left(\omega \right)+\alpha^{\left(3\right)}\left(\omega ,I\right),$$

where $\alpha^{\left(1\right)}$ is given by,

(7) $$\alpha^{\left(1\right)}\left(\omega \right)=\omega \sqrt{\frac{\mu }{\varepsilon }}\frac{\hbar \;\Gamma_{\mathrm{if}}\;{\left|M_{\mathrm{fi}}\right|}^{2}\;\sigma }{{\left(E_{\mathrm{fi}}-\hbar \omega \right)}^{2}+{\left(\hbar \;\Gamma_{\mathrm{if}}\right)}^{2}}$$

and the expression of third order AC is written as:

(8) $$\begin{array}{cc} \alpha^{\left(3\right)}\left(\omega ,I\right)= & -\omega \sqrt{\frac{\mu_{}}{\varepsilon }}\left(\frac{I}{2\varepsilon_{0}n_{r}c}\right)\frac{4\sigma \hbar \Gamma_{\mathrm{if}}{\left|M_{\mathrm{fi}}\right|}^{4}}{{\left[{\left(E_{\mathrm{fi}}-\hbar \omega \right)}^{2}+{\left(\hbar \Gamma_{\mathrm{if}}\right)}^{2}\right]}^{2}}\\ & \left[1-\frac{{\left(M_{\mathrm{ff}}-M_{ii}\right)}^{2}}{4{\left|M_{\mathrm{fi}}\right|}^{2}}\left(\frac{3E_{\mathrm{fi}}^{2}-4\hbar \omega E_{\mathrm{fi}}+\hbar^{2}\left(\omega^{2}-\Gamma_{\mathrm{if}}^{2}\right)}{E_{\mathrm{fi}}^{2}+{\left(\hbar \Gamma_{\mathrm{if}}\right)}^{2}}.\right)\right] \end{array}$$

In these conditions, the RICs written as

(9) $$\frac{\Delta n(\omega )}{n_{r}}=\frac{\Delta n^{\left(1\right)}\left(\omega \right)}{n_{r}}+\frac{\Delta n^{\left(3\right)}\left(\omega \right)}{n_{r}},$$

where

(10) $$\frac{\Delta n^{\left(1\right)}\left(\omega \right)}{n_{r}}=\frac{1}{2\varepsilon_{0}n_{r}^{2}}\frac{\sigma {\left|M_{\mathrm{fi}}\right|}^{2}\left(E_{\mathrm{fi}}-\hbar \omega \right)}{{\left(E_{\mathrm{fi}}-\hbar \omega \right)}^{2}+{\left(\hbar \Gamma_{\mathrm{if}}\right)}^{2}}$$

and

$$\begin{array}{ccc} \frac{\Delta n^{\left(3\right)}\left(\omega ,I\right)}{n_{r}} & = & -\left(\frac{\mu_{}cI\sigma {\left|M_{\mathrm{fi}}\right|}^{4}}{\varepsilon_{0}n_{r}^{3}}\frac{\left(E_{\mathrm{fi}}-\hbar \omega \right)}{{\left[{\left(E_{\mathrm{fi}}-\hbar \omega \right)}^{2}+{\left(\hbar \Gamma_{\mathrm{if}}\right)}^{2}\right]}^{2}}\right)\\ & & \left[1-\frac{{\left(M_{\mathrm{ff}}-M_{ii}\right)}^{2}}{4{\left|M_{\mathrm{fi}}\right|}^{2}\left(E_{\mathrm{fi}}^{2}+{\left(\hbar \Gamma_{\mathrm{if}}\right)}^{2}\right)}\left(E_{\mathrm{fi}}\left(E_{\mathrm{fi}}-\hbar \omega \right)-{\left(\hbar \Gamma_{\mathrm{if}}\right)}^{2}-{\left(\hbar \Gamma_{\mathrm{if}}\right)}^{2}\frac{\left(2E_{\mathrm{fi}}-\hbar \omega \right)}{\left(E_{\mathrm{fi}}-\hbar \omega \right)}\right)\right] \end{array}$$

where $E_{\mathrm{fi}}=E_{f}-E_{i}$ denotes the energy transition between an initial state (i) and a finale state (f), $M_{\mathrm{fi}}=e<\Psi_{f}\left|x\right|\Psi_{i}>$ is the electric dipole moment of the transition from the *i* states to *f* states. We recall that the parameters used above are: *c* for the speed of light in a vacuum, $\sigma_{s}$ is the electron density related to the occupied volume by the relation. *I* is the intensity of the incident electromagnetic radiation, ω is the angular frequency of the laser radiation, μ is the permeability of the system, $n_{r}$ is the relative refractive index of semiconductor, $\varepsilon_{0}$ is the permittivity of free space. $\Gamma_{\mathrm{fi}}$ is the line width and also recognized as the non-diagonal matrix element defined as the inverse of the relaxation time $\tau_{\mathrm{fi}}$ and known as the relaxation rate of initial and final states.

Whenever an electromagnetic field $E\left(t\right)=E_{0}e^{j(\omega t)}+E_{0}e^{-j(\omega t)}$, with frequency ω interacts with the system, there are changes in the polarization which has both linear and non-linear components as the electric field can be expanded using power series [63], therefore we have optical rectification (OR), second harmonics generation (SHG), third harmonic generation (THG) and so on. Second and third-order susceptibilities help to calculate SHG and THG coefficients that provide total information for the optical response of the system along with ACs and RICs. We recall that the electronic polarization of the *n*th order on E(t) can be expressed as [41]:

(11) $$P^{n}\equiv \varepsilon_{0}[\chi^{\left(1\right)}E\left(t\right)+\chi^{\left(2\right)}E^{2}\left(t\right)+\chi^{\left(3\right)}E^{3}\left(t\right)+\ldots ].,$$

where $\varepsilon_{0}$ is the permittivity of the free space, $\chi^{\left(1\right)},\chi^{\left(2\right)},\chi^{\left(3\right)}$ are the linear, second-order and third-order susceptibilities respectively and $E\left(t\right),E^{2}\left(t\right),E^{3}\left(t\right)$ linear, second-order and third-order optical electric field respectively. Using the compact density matrix method and an iterative procedure we can obtain the formula for the SHG and THG coefficients [64,65,66].

(12) $$\chi_{2\omega }^{\left(2\right)}=\frac{\sigma }{\varepsilon_{0}}\frac{M_{12}M_{23}M_{31}}{\left(\hbar \omega -E_{21}-j\hbar \Gamma_{21}\right)\left(2\hbar \omega -E_{31}-j\hbar \Gamma_{31}\right)}$$

and

(13) $$\chi_{3\omega }^{\left(3\right)}=\frac{\sigma }{\varepsilon_{0}}\frac{M_{12}M_{23}M_{34}M_{41}}{\left(\hbar \omega -E_{21}-j\hbar \Gamma_{21}\right)\left(2\hbar \omega -E_{31}-j\hbar \Gamma_{31}\right)\left(3\hbar \omega -E_{41}-j\hbar \Gamma_{41}\right)},$$

where interstate damping terms are as follows: $\Gamma_{21}=\Gamma_{0}\;,\Gamma_{31}=\Gamma_{0}/2,\;\Gamma_{41}=\Gamma_{0}/3$. The product of different elements $M_{12}M_{23}M_{34}M_{41}$ has an important effect on the generation of THG coefficients.

## 3. Results and Discussion

In our calculation, we assumed a SiGe/Si nanostructure and we used the following parameters: For η<0.85, the $Si_{1-\eta }Ge_{\eta }$ alloys are considered to be an Si-like material. The parameters used in the numerical calculations for SiGe are as follows: $\sigma =2.8\times 10^{25}$ m^−3^, the electron density, $n_{r}\left(\eta =0.3\right)=3.55$ represents the refractive index of the QD, ε(η=0.3)=13.05 is the static dielectric constant, $\Gamma_{\mathrm{if}}=0.38$ ps^−1^ is the inverse of the relaxation time material and $I=2\times 10^{7}$ W/m^2^ is the intensity of the incident electromagnetic field [59,67]. The effective mass of conductivity $m_{SiGe}^{*}=0.26m_{0}$, where $m_{0}$ is the mass of free electrons.

Based on the theoretical methods presented in the previous section, we have calculated the effect of confining parameters such as height (h) and radius (R) of prolate and oblate shapes on the energy levels, dipole matrix (DM) elements. Finally, we have calculated the ACs, RICs and the second and third harmonic generations, showing the effect of various parameters on these properties.

In such shapes, the solution of the Schrdinger equation is almost impossible, so Equation (1) is solved numerically by FEM (COMSOL Multiphysics 5.4 software [68]). The Schrdinger equation was put in the form of the general partial differential equation (PDE). The eigenvalue solver was used with a zero-flux boundary condition (B1,B5), the Dirichlet boundary condition (ψ=0) is used, and the six lateral boundaries (B2, B3, B4, B6, B7 and B8), the Neumann boundary condition $\hat{n}.\vec{▿}.\Psi =0$ is used. The boundary condition of $\hat{n}.\left(▿.\Psi /m_{SiGe}^{*}\right)=\hat{n}.\left(▿.\Psi /m_{Si}^{*}\right)$ is used for the interface limits of Si and SiGe due to the potential finiteness. In our calculations, we used an extra refined mesh with a number of degrees of freedom ∼14,400. The thickness WL was also set at 0.5 nm according to experimental reports [69].

First of all, we determine the evolution of the energy band gap at the zero electric field as a function of the height and radius of oblate and prolate QD, respectively.

**Figure 2:**
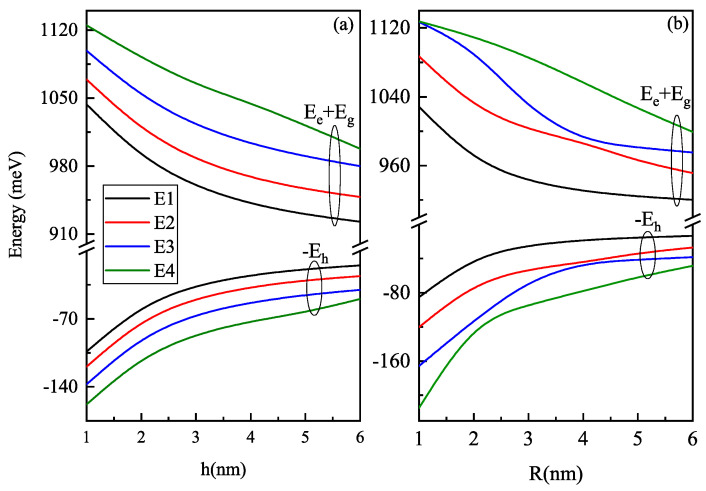
Variation of four low lying energy levels as a function of the (**a**) oblate QD height (with R = 7 nm) and (**b**) prolate QD R (with h = 7 nm) without electric field i.e., F=0 kV/cm for electron and hole states.

Figure 2 shows four low lying energy levels for electron and hole states as a function of the (a) oblate QD height (with R = 7 nm) and (b) prolate QD R (with h = 7 nm) without electric field, that is, F=0 kV/cm. The bandgap for both oblate and prolate QD decreases with an increase in size as the QD suffers weak confinement. The behavior of $E_{2}$ and $E_{3}$ energy levels is exchanged around 4 nm and of $E_{3}$ and $E_{4}$ around 2 nm as the variety of hole states shows the avoided crossing region. The avoided crossing occurs in the system to improve stability. It is a benchmark of the electric field effect. The curves are more parallel with the change in height h of the oblate QD but less parallel for the change in the prolate QD radius R, as the volume of oblate/prolate QD is proportional to $R^{2}\;h$. The linear dependence of volume h makes less of a change in confinement compared to the R changes, so the confinement is larger in oblate QD than in prolate QD, which then affects the energy levels more for change in the R case (prolate QD) than for the change in the h case (oblate QD).

### 3.1. With Lateral Electric Field (In x-Direction)

**Figure 3:**
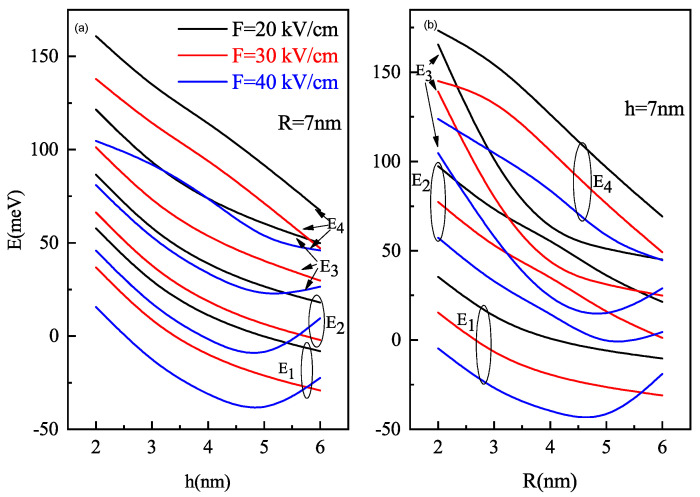
Ground and excited state energies as a function of the (**a**) oblate QD height h and (**b**) prolate QD radius R for different values of parallel electric field F=20, 30 and 40 kV/cm.

To understand the dynamics of the system with the size of the QD and electric field strengths, we need to understand the energy levels variation of semi oblate/prolate QD with height/radius with different lateral electric field strengths. For the same, we have obtained Figure 3.

As with the decrease in QD size, the confinement strength increases due to a reduction in the volume. But by increasing the intensity of the electric field increase in electric field strength, the energy for respective states of all levels decreases. We have a competition of two antagonistic effects(confinement and stark effect). For the h ≃ 5 nm in oblate QD with R = 7 nm and R ≃ 5 nm in prolate QD with h = 7 nm, the energy of levels starts to increase as the weaker confined region, electric field effect is more dominant. Compared to oblate QD, the prolate QD levels have higher energy for the same field strength. It is due to the larger volume of oblate QD in contrast to prolate QD for the same combination of h and R values that lead to weaker confinement in oblate QD compared to prolate QD.

**Figure 4:**
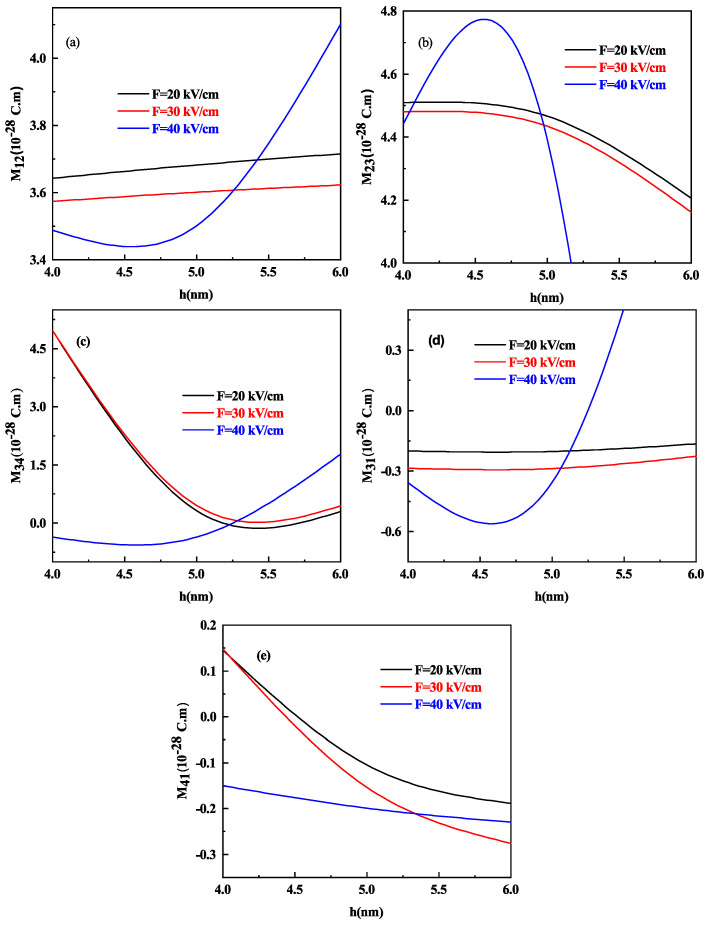
The DM elements (**a**) $M_{12}$, (**b**) $M_{23}$, (**c**) $M_{34}$, (**d**) $M_{31}$, and (**e**) $M_{41}$ as a function of oblate QD height h for R = 7 nm and for different values of lateral electric field F=20, 30 and 40 kV/cm respectively.

The optical response of the system is well known to be dominated by transition DM elements. To analyze the effect of the height in oblate shape, we present Figure 4a–e, that shows the variation of different transition DM elements that is $M_{12}$, $M_{23}$, $M_{34}$, $M_{31}$, and $M_{41}$ as a function of oblate QD height h with R = 7 nm for different values of electric field F=20, 30 and 40 kV/cm respectively.

At 40 kV/cm strength of the electric field, the behavior of all DM elements gets increased (decreased) as compared to F=20 kV/cm and 30 kV/cm. $M_{12}$ and $M_{31}$ DM element increases with an increase in the height of oblate QD in general (see Figure 4a,d) whereas $M_{23},M_{34}$ and $M_{41}$ transitions shows a decrease in value as the height of oblate QD increases as shown in Figure 4b,c,e respectively, this is on account of increase in energy. It is worth noting that the DM element $M_{34}$ (Figure 4c) decreases with increasing oblate QD height but at 40 kV/cm strength of the electric field, after a slight decrease, it starts increasing as the energy of $E_{3}$ and $E_{4}$ levels also increase after a 5 nm height of oblate QD as shown in Figure 3a.

**Figure 5:**
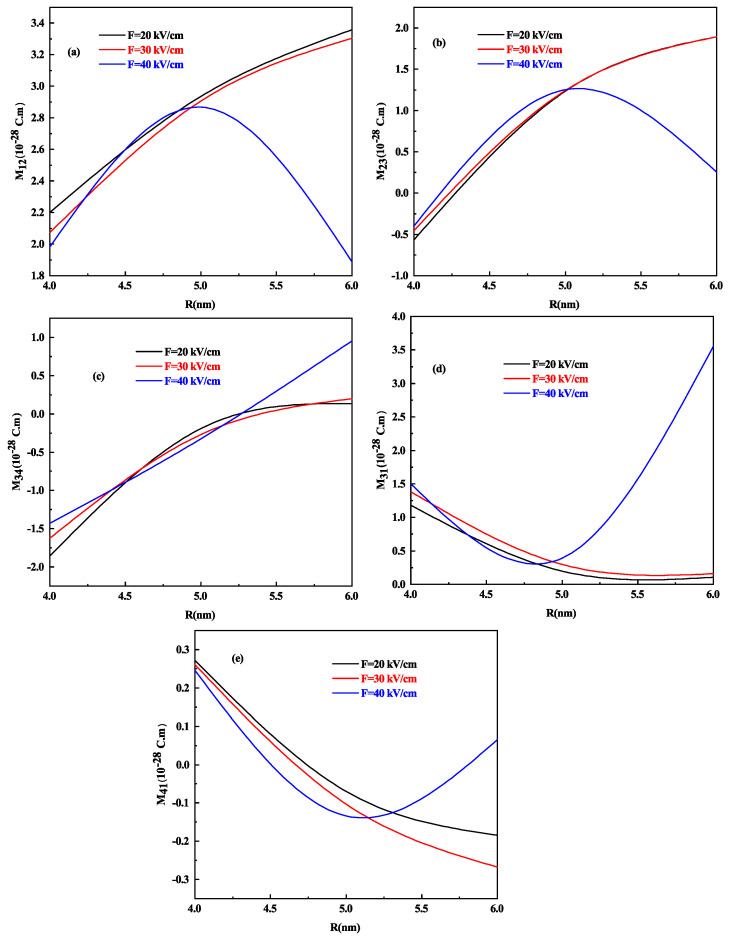
The DM elements (**a**) $M_{12}$, (**b**) $M_{23}$, (**c**) $M_{34}$, (**d**) $M_{31}$, and (**e**) $M_{41}$ as a function of prolate QD Radius R for h = 7 nm and for different values of the lateral electric field F=20, 30 and 40 kV/cm respectively.

Let us analyze the effect of the lateral dimension on the DM elements of prolate shaped QD we draw Figure 5a–e which shows the variation of different transition DM elements $M_{12}$, $M_{23}$, $M_{34}$, $M_{31}$, and $M_{41}$ as a function of prolate QD radius R with h = 7 nm for different values of electric field F=20, 30 and 40 kV/cm respectively. Here, too, at 40 kV/cm strength of the electric field, the behavior of all DM elements is increased (decreased) as compared to F=20 kV/cm and 30 kV/cm. $M_{12},\;M_{23}$, and $M_{34}$ DM element that is Figure 5a–c, increases with the increase in the radius of prolate QD in general, whereas $M_{31}$ and $M_{41}$ transitions that is Figure 5d,e shows a decrease in value as the radius of prolate QD increases, this is on account of the increase in energy only for F=20 kV/cm and 30 kV/cm, whereas the reverse is observed for 40 kV/cm. It is because the energy of the levels also increases after a 5 nm radius of the prolate QD as shown in Figure 3b.

We recall that the dipole matrix element is system-specific, hence their behavior cannot be predicted. We have solved the dipole matrix element for a different system of conical quantum dots [66] and verified their dipole matrix elements.

It is crucial to note that the different behavior at 40 kV/cm for all the DM elements for both oblate and prolate QD. The electric field acts as an additional repulsive potential that changes the behavior of the wavefunction. At a high electric field strength in oblate QD, when we weaken the confinement say for the $M_{12}$ DM element that is Figure 4a, $M_{12}$ first increases and then starts decreasing. The maxima of $M_{ij}$ is the outcome of the competition between the confinement potential and electric field potential term. However, in the prolate QD case, $M_{12}$ increases with weak confinement and decreases with electric field intensity at low electric fields, but after a small increase, it begins to decrease and then increases when the confinement is reduced at high electric fields. We see this disparity in the behavior of $M_{ij}$ for oblate and prolate QD since the volume decreases quadratically with radius R and linearly with height h. The competition between the effective potential by confinement and electric field controls the behavior of the wave function. When the confinement is very small, the system will act as a free particle with no effect from the electric field. We cannot predict the behavior of dipole matrix elements at the different electric fields, so we can say that the electric field can be used as a controlling parameter. When we have large DM elements, the response towards the other parameters, such as towards different optical properties, will be high.

**Figure 6:**
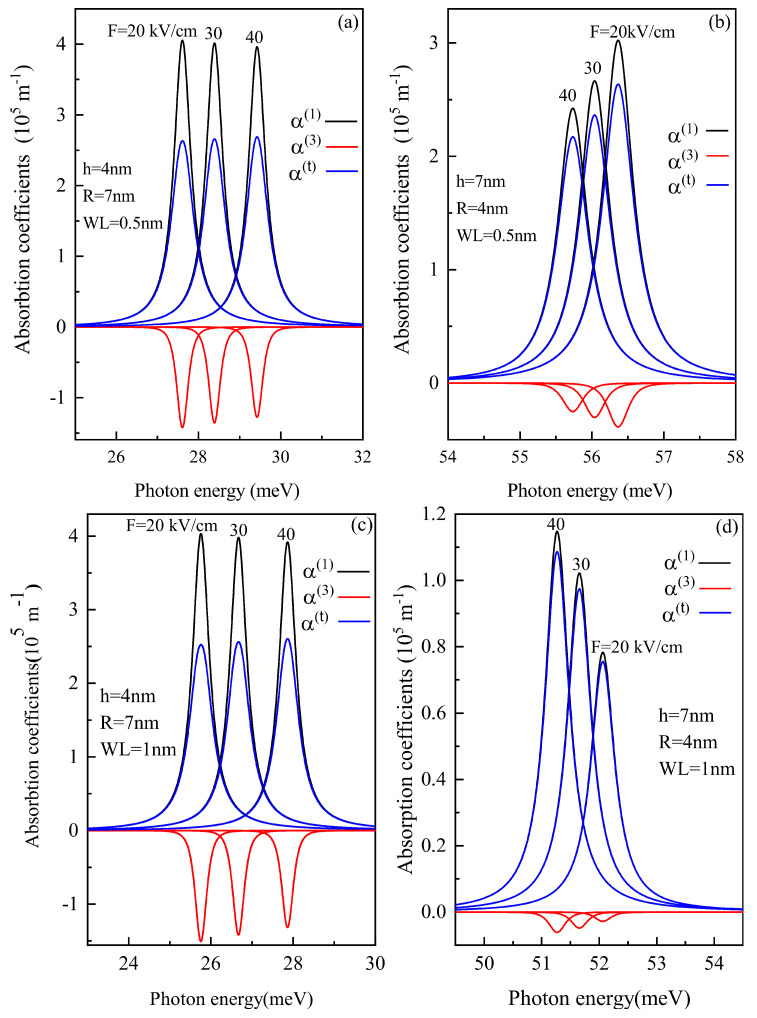
Linear, non-linear and total absorption coefficients as a function of the photon energy for (**a**) oblate height R = 4 nm with radius R = 7 nm at WL thickness 0.5 nm (**b**) prolate QD with R = 4 nm, h = 7 nm with WL thickness = 0.5 nm, (**c**) oblate QD with R = 4 nm, R = 7 nm with WL thickness = 1 nm, and (**d**) prolate QD with R = 4 nm, h = 7 nm with WL thickness = 1 nm for different values of the lateral electric field F=20, 30 and 40 kV/cm.

The optical ACs have a sharp peak where the incident light energy is equal to the transition energy between the two levels. In Figure 6a,c, the linear, non-linear (third-order) and total AC for the oblate QD of height 4 nm and radius 7 nm with a WL thickness of 0.5 nm and 1 nm, respectively, is shown for different electric field strengths. With an increase in electric field amplitude, a blue shift occurs in AC due to the decrease in energy with an electric field( see Figure 3a). The amplitude of AC is proportional to $|M_{fi}{|}^{2}$, which, from Figure 4a, it is clear that with an increase in the electric field, the value of $M_{12}$ element increases.

Figure 6b,d shows the linear, non-linear (third-order) and total AC for the prolate QD of radius 4 nm and height 7 nm with a WL thickness of 0.5 nm and 1 nm respectively, for three electric field strengths of 20, 30 and 40 kV/cm. As shown in Figure 6b,d, blueshift occurs for an increase in electric field strength, this effect is easily explained by Figure 3b and Figure 5a. However, Figure 6b, shows a decrease in amplitude while Figure 6d shows an increase in amplitude with an increase in electric field strength. This explains that the electric field’s impact on optical AC will not be the same for a particular shape for different WL thicknesses. As with the increase in WL, a change in energy levels occurs, which also impacts the DM elements.

To analyze the effect of the WL thickness on the optical properties, we determined the linear, non-linear (third-order) and total AC as a function of photon energy with different electric field values at different WL thicknesses. With an increase in WL thickness, redshift is noticed in the optical AC peaks as the transition energy decreases with an increase in the WL thickness.

**Figure 7:**
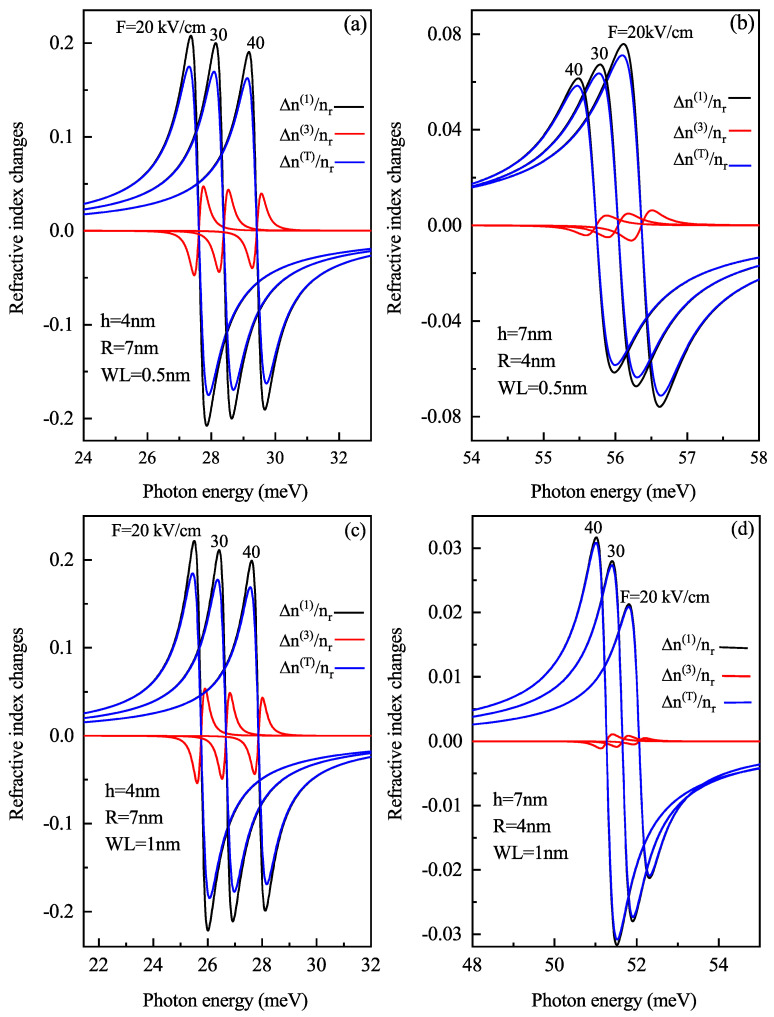
Linear, non-linear and total refractive index changes as a function of the photon energy for (**a**) oblate height R = 4 nm with radius R = 7 nm at WL thickness 0.5 nm (**b**) prolate QD with R = 4 nm, h = 7 nm with WL thickness = 0.5 nm, (**c**) oblate QD with R = 4 nm, R = 7 nm with WL thickness = 1 nm, and (**d**) prolate QD with R = 4 nm, h = 7 nm with WL thickness = 1 nm for different values of the lateral electric field F=20, 30 and 40 kV/cm.

Figure 7 demonstrates the linear, third-order and total RICs as a function of the photon energy for (a) oblate height h = 4 nm with radius R = 7 nm at a WL thickness 0.5 nm (b) prolate QD with R = 4 nm, h = 7 nm with a WL thickness = 0.5 nm, (c) oblate QD with h = 4 nm, R = 7 nm with a WL thickness = 1 nm, and (d) prolate QD with R = 4 nm, h = 7 nm with a WL thickness = 1 nm for different values of electric field F=20, 30 and 40 kV/cm. Similar to Figure 6, here too with an increase in electric field amplitude, a blue shift occurs in RIC due to the decrease in energy with an electric field as shown in Figure 3a. Figure 7b,d, shows blueshift for an increase in electric field strength but Figure 7b shows a decrease in amplitude while Figure 7d shows an increase in amplitude with an increase in electric field strength. With an increase in WL thickness, redshift is noticed in optical RIC peaks as the transition energy decreases with an increase in the WL thickness.

**Figure 8:**
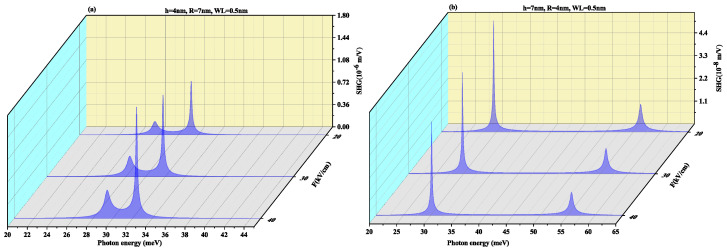
Second harmonic generation as a function of the photon energy for (**a**) oblate height R = 4 nm with radius R = 7 nm at WL thickness 0.5 nm (**b**) prolate QD with R = 4 nm, h = 7 nm with WL thickness = 0.5 nm for different values of the lateral electric field F=20, 30 and 40 kV/cm.

SHG as a function of the photon energy for (a) oblate height R = 4 nm with radius R = 7 nm at a WL thickness of 0.5 nm (b) prolate QD with R = 4 nm, h = 7 nm with WL thickness = 0.5 nm for different values of electric field F=20, 30 and 40 kV/cm, is shown in Figure 8. In oblate QD (Figure 8a), with an increase in the electric field strength, the two resonance peaks tend to merge, whereas in Figure 8b the resonance peaks are distant. Blueshift is also visible in peaks with a rise in amplitude on increasing the electric field strength. In Figure 8b prolate QD, no shift is observed in peaks with an increase in the strength of the electric field, however, the amplitude of peaks reduces with an increase in strength of the electric field. The order of the SHG for SiGe is close to the GaAs QD and CdS QD [70,71], that is, $10^{-7}$ m/V.

**Figure 9:**
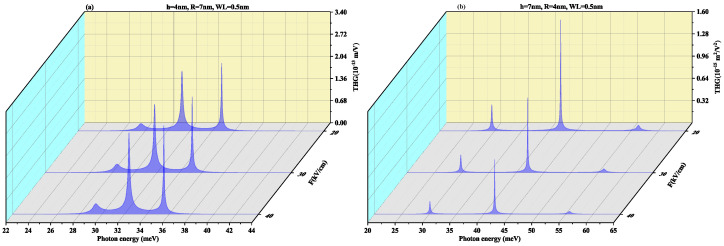
Third harmonic generation as a function of the photon energy for (**a**) oblate height R = 4 nm with radius R = 7 nm at WL thickness 0.5 nm (**b**) prolate QD with R = 4 nm, h = 7 nm with WL thickness = 0.5 nm for different values of lateral electric field F=20, 30 and 40 kV/cm.

Figure 9 displays THG as a function of the photon energy for (a) oblate height R = 4 nm with radius R = 7 nm at WL thickness 0.5 nm (b) prolate QD with R = 4 nm, h = 7 nm with WL thickness = 0.5 nm for different values of electric field F=20, 30 and 40 kV/cm. In oblate QD (Figure 9a), with an increase in the electric field strength, the two resonance peaks tend to merge with increasing the electric field strength whereas Figure 9b shows that the resonance peaks are distant. Blueshift is also visible in the pinnacle with a rise in magnitude but in Figure 9b prolate QD, no shift is observed in peaks with an increase in strength of the electric field. However, the amplitude of peaks suffers a decrease with an increase in strength of the electric field. THG coefficients for CdS [71] QD ($10^{-17}$ m^2^/V^2^) and for the SiGe case are near to the GaAs/AlAs case ($10^{-14}$ m^2^/V^2^) as reported by Reference [72].

**Figure 10:**
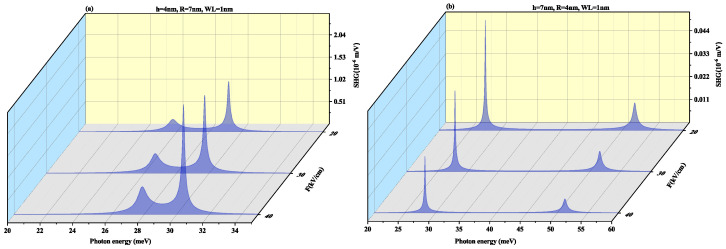
Second harmonic generation as a function of the photon energy for (**a**) oblate height R = 4 nm with radius R = 7 nm at WL thickness 1 nm (**b**) prolate QD with R = 4 nm, h = 7 nm with WL thickness = 1 nm for different values of lateral electric field F=20, 30 and 40 kV/cm.

**Figure 11:**
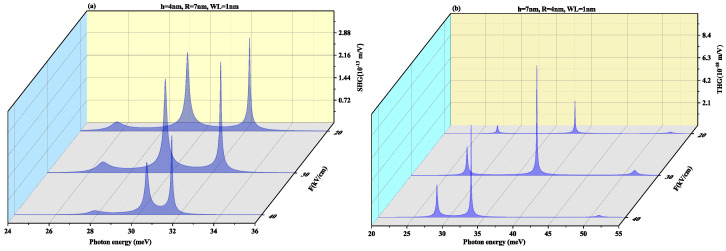
Third harmonic generation as a function of the photon energy for (**a**) oblate height R = 4 nm with radius R = 7 nm at WL thickness 1 nm (**b**) prolate QD with R = 4 nm, h = 7 nm with WL thickness = 1 nm for different values of lateral electric field F=20, 30 and 40 kV/cm.

To observe the effect of the wetting layer in the QD geometry, we have plotted the SHG and THG at a WL thickness of 1 nm for both oblate (h = 4 nm and R = 7 nm) and prolate (h = 7 nm and R = 4 nm) QD in Figure 10 and Figure 11, respectively. On increasing the WL thickness to 1 nm, the redshift arises in resonance peaks for SHG and THG. With an increase in an electric field, the same results are achieved for SHG with a slight increase in magnitude as for Figure 8 and THG results are similar to Figure 9 with a decrease in amplitude, with an increase in WL thickness.

### 3.2. With Electric Field (In z-Direction) Perpendicular to Wl

We have recognized that the electric field affects the system’s optical response, but it is also the field’s direction of operation that defines the extent of change in the response. Therefore, to compare the effect of the lateral and perpendicular field, we have computed the variation of the different optical properties for the perpendicular electric field as well. Let us analyze the behavior of the optical properties of SiGe/Si QD when the electric field is applied perpendicular to WL. We start with an analysis of the energy spectra corresponding to the first four low lying states.

**Figure 12:**
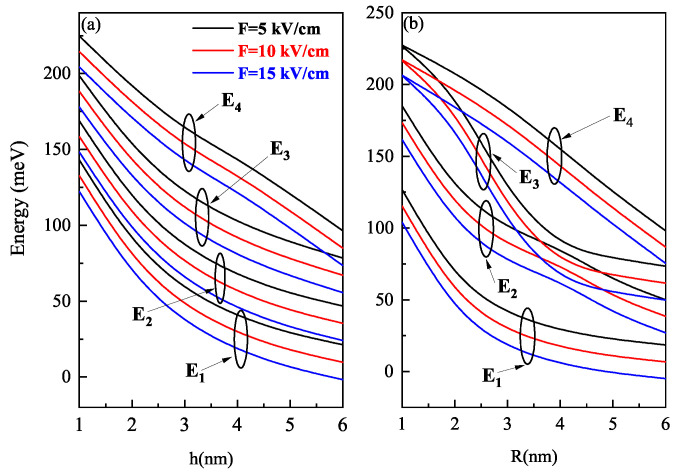
Ground and excited state energies as a function of the (**a**) oblate QD height (with R = 7 nm) and (**b**) prolate QD R (with h = 7 nm) for different values of the perpendicular electric field at F=5,10 and 15 kV/cm.

Figure 12 shows the variation of energy of states with electric field strength. The energy of four low-lying states in both oblate and prolate QD decreases as the electric field strength increases. It is due to the increase in the contribution of the repulsion term. It is important to note here that the $E_{2}$ and $E_{3}$ levels in prolate QD avoided crossing around R = 4 nm with an increase in the electric field strength of the prolate QD.

**Figure 13:**
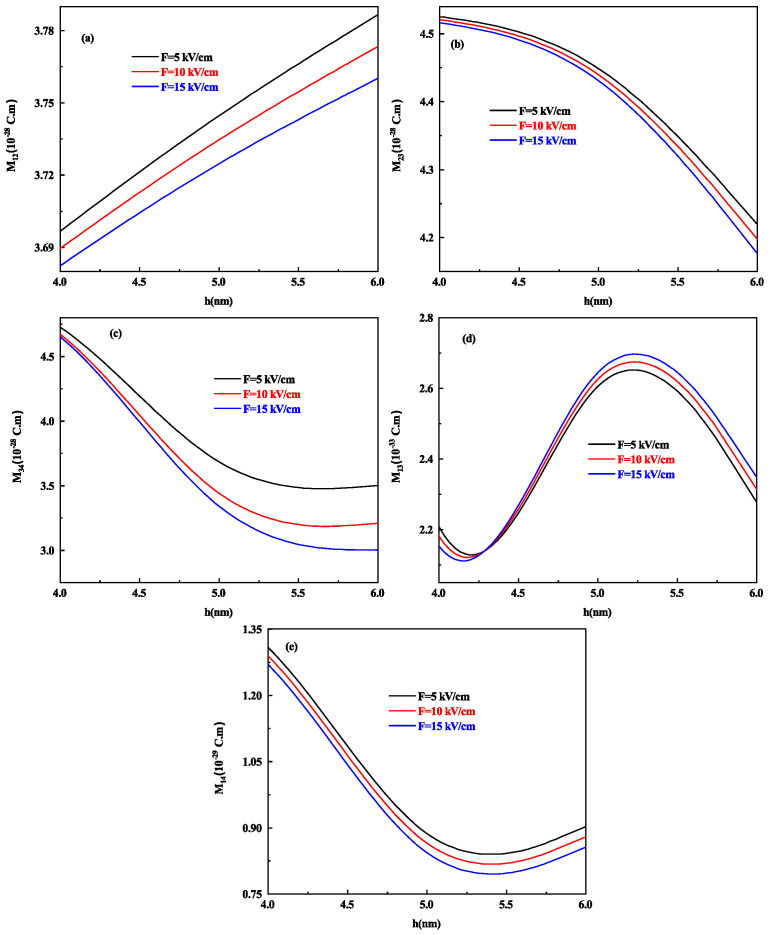
The DM elements (**a**) $M_{12}$, (**b**) $M_{23}$, (**c**) $M_{34}$, (**d**) $M_{31}$, and (**e**) $M_{41}$ as a function of oblate QD height h with R = 7 nm for different values of the perpendicular electric field F=5,10 and 15 kV/cm respectively.

**Figure 14:**
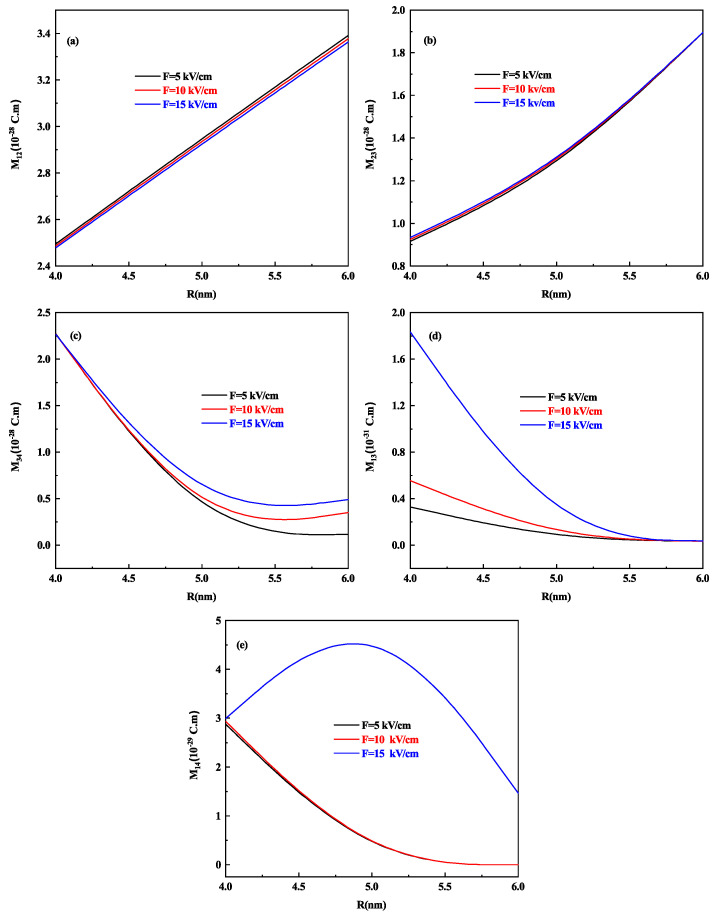
The DM elements (**a**) $M_{12}$, (**b**) $M_{23}$, (**c**) $M_{34}$, (**d**) $M_{31}$, and (**e**) $M_{41}$ as a function of prolate QD radius R with h = 7 nm for different values of the perpendicular electric field F=5,10 and 15 kV/cm respectively.

The variation of different DM elements that is $M_{12}$, $M_{23}$, $M_{34}$, $M_{31}$, and $M_{41}$ with the height of oblate QD with a radius of 7 nm at the different electric field strength is shown in Figure 13a–e. $M_{12}$ (Figure 13a) shows an almost linear variation with an increase in size and, with an increase in field strength, this rate decreases. $M_{23}$ that is Figure 13b shows a decrease with an increase in height of the oblate QD. $M_{34}$ and $M_{41}$ (Figure 13c,e) shows similar behavior of a decrease in value. $M_{31}$ in Figure 13d shows a peak at a 5.25 nm height of oblate QD and on either side it decreases. The change in the DM element of prolate QD is large compared to oblate QD as an example, in Figure 13a, $M_{12}$ for oblate QD of radius 7 nm suffers a change of approx. 0.09 with the change in height from 4 nm to 6 nm, while 7 nm height prolate QD (Figure 14a) sufferers a change of around 0.9 for the change in radius from 4 nm–6 nm. This is due to the large surface-to-volume ratio in the case of oblate QD in comparison with prolate QD, as the volume is directly proportional to $R^{2}h$.

For prolate QD of height 7 nm, the variation of DM elements that is $M_{12}$, $M_{23}$, $M_{34}$, $M_{31}$, and $M_{41}$ is shown in Figure 14a–e respectively. $M_{12}$ and $M_{23}$ show a near-linear variation with an increase in the radius of prolate QD in Figure 14a,b. $M_{34}$, $M_{31}$ and $M_{41}$ in Figure 14c–e show a decrease with an increase in the radius of prolate QD, and with an increase in electric field strength, the value of DM elements increases.

The transition matrix elements evaluated are defined as $≺\Psi_{i}\left|er\right|\Psi_{j}≻$, as with the electric field wavefunction changes and with the change in radius (R) and height (h), the same phenomenon happens, so that is why the matrix elements change with either electric field or change in dimension.

**Figure 15:**
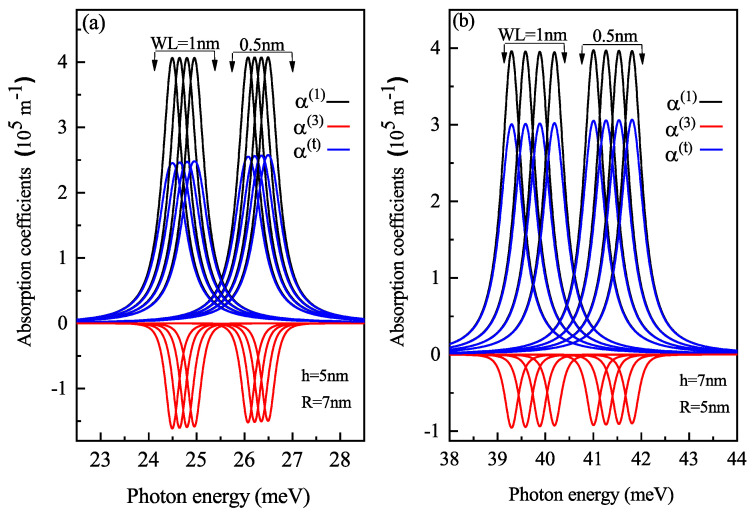
Linear, non-linear and total absorption coefficients as a function of the photon energy for (**a**) oblate height h = 5 nm with radius R = 7 nm at WL thickness 0.5 nm and 1 nm (**b**) prolate QD with R = 5 nm, h = 7 nm with WL = 0.5 nm and 1 nm, at four perpendicular electric field strengths of F=0,5,10 and 15 kV/cm from left to right respectively.

**Figure 16:**
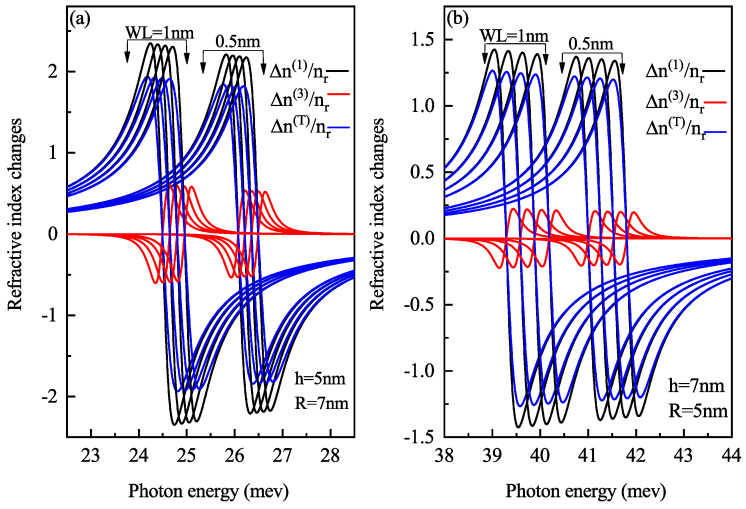
Linear, non-linear and total refractive index changes as a function of the photon energy for (**a**) oblate height h = 5 nm with radius R = 7 nm at WL thickness 0.5 nm and 1 nm (**b**) prolate QD with R = 5 nm, h = 7 nm with WL = 0.5 nm and 1 nm, at four perpendicular electric field strengths of F=0,5,10 and 15 kV/cm from left to right respectively.

Figure 15 shows the AC variation with the energy of laser radiation for oblate QD with height 5 nm and radius 7 nm and prolate QD with height 7 nm and radius 5 nm with a WL thickness of 0.5 nm and 1 nm at four perpendicular electric field strengths of F=0,5,10 and 15 kV/cm from left to right, respectively. Redshift is observed with an increase in WL thickness, with an increase in amplitude. With an increase in electric field strength, $M_{12}$ element decreases, hence blueshift is observed with a decrease in magnitude with an increase in electric field strength. The resonance absorption peak is at higher laser energy in prolate QD compared to oblate QD because the confinement is strong in prolate QD compared to oblate QD as this set of h and R has a large volume in oblate QD, which leads to weak confinement. The surface-to-volume ratio defines the confinement. For oblate QD of h = 5 nm and R = 7 nm, the S/V ratio is 3.97 and for prolate QD of h = 7 nm and R = 5 nm, the S/V ratio is 1.83. For a large S/V ratio, the confinement effect is weak. Corresponding results are obtained for RICs for oblate and prolate QD as shown in Figure 16. RIC follows the same behavior as AC i.e., blueshift with increasing electric field and redshift with WL thickness. The shift in the resonance peak is almost linear for both prolate and oblate QD as the transition matrix element $M_{12}$ is linear with the change in electric field strength.

**Figure 17:**
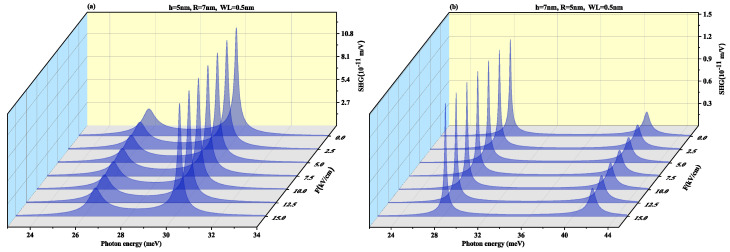
Second harmonic generation as a function of the photon energy for (**a**) oblate height h = 5 nm with radius R = 7 nm at WL thickness 0.5 nm (**b**) prolate QD with R=5 nm, h = 7 nm with WL thickness = 0.5 nm for different perpendicular electric field strengths from *F* = 0 to 15 kV/cm.

**Figure 18:**
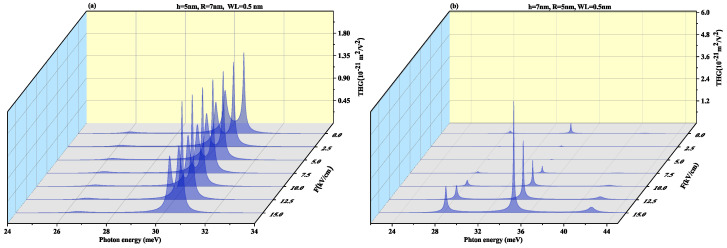
Third harmonic generation as a function of the photon energy for (**a**) oblate height h = 5 nm with radius R = 7 nm at WL thickness 0.5 nm (**b**) prolate QD with R = 5 nm, h = 7 nm with WL thickness = 0.5 nm for different perpendicular electric field strengths from F=0 to 15 kV/cm.

Figure 17 and Figure 18 displays SHG and THG as a function of the photon energy for (a) oblate height h = 5 nm with radius R = 7 nm at WL thickness 0.5 nm (b) prolate QD with R = 5 nm, h = 7 nm with WL = 0.5 nm for different perpendicular electric field strengths from *F* = 0 to 15 kV/cm. Blueshift is observed with an increase in an electric field with a decrease in amplitude for oblate QD of height 5 nm with radius 7 nm for both SHG and THG. As the $M_{12}$, $M_{23}$, $M_{34}$ and $M_{14}$ show a decrease while $M_{13}$ shows an increase with the increase in electric field strength, so an overall decrease in peaks amplitude is observed and δE increases with increase in electric field strength as visible from Figure 12a. The prolate QD with height 7 nm and radius 5 nm with WL thickness of 0.5 nm we observe an increase in amplitude in SHG. An increase in electric field strength shows a blueshift in THG resonance peaks with an increase in peak amplitude. As the $M_{13}$, $M_{23}$, $M_{34}$ and $M_{14}$ show an increase while $M_{12}$ shows a decrease with the increase in electric field strength, so an overall increase in peaks amplitude is observed and δE increases with increase in electric field strength as visible from Figure 12b.

**Figure 19:**
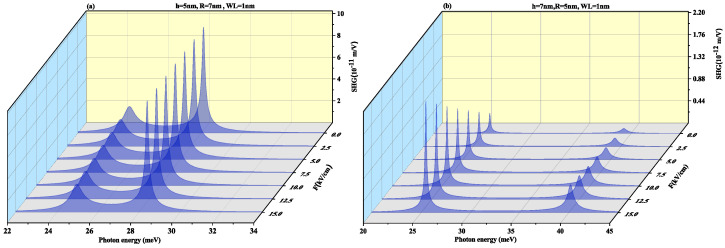
Second harmonic generation as a function of the photon energy for (**a**) oblate height h = 5 nm with radius R = 7 nm at WL thickness 1 nm (**b**) prolate QD with R = 5 nm, h = 7 nm with WL thickness = 1 nm for different perpendicular electric field strengths from F=0 to 15 kV/cm.

**Figure 20:**
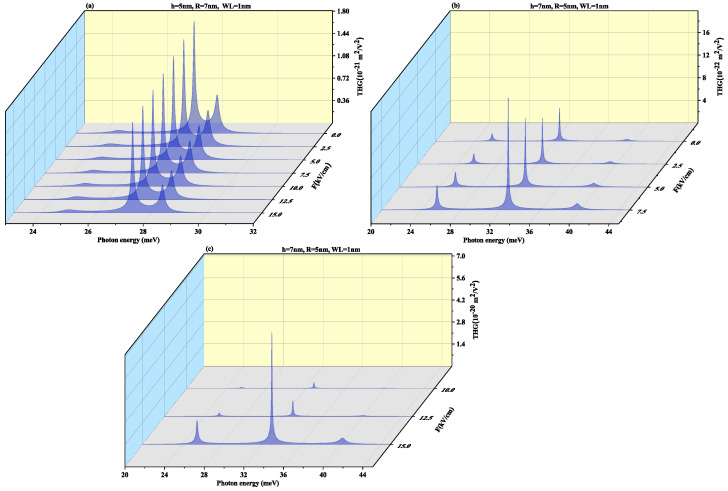
Third harmonic generation as a function of the photon energy for (**a**) oblate height h = 5 nm with radius R = 7 nm at WL thickness 1 nm (**b**) prolate QD with R = 5 nm, h = 7 nm with WL thickness = 1 nm for different perpendicular electric field strengths from F=0 to 15 kV/cm.

Figure 19 and Figure 20 demonstrate SHG and THG as a function of the photon energy for (a) oblate height h = 5 nm with radius R = 7 nm at a WL thickness of 1 nm (b) prolate QD with R = 5 nm, h = 7 nm with WL thickness = 1 nm for different perpendicular electric field strengths from F=0 to 15 kV/cm. Here too, blueshift is observed with an increase in an electric field with a decrease in amplitude for oblate QD for SHG and THG while prolate QD with height 7 nm and radius 5 nm with WL thickness of 1 nm show blueshift with an increase in amplitude for increasing electric field strength. With an increase in WL thickness, that is, 1 nm redshift in resonance peaks.

From the figures, it is clear that the electric field direction is an important factor for controlling optical response. The SHG and THG for the lateral electric field are approximately 1000 times larger compared to the perpendicular electric field.

## 4. Conclusions

The lateral and perpendicular electric field sway on the optoelectronic properties is researched for SiGe semi-oblate and semi-prolate QDs with SiGe WL in the environment of the Si matrix with 30% Ge composition. Our analysis showed that the redshift is caused by the energy levels of the electric field and increased size (height or radius) of the QD. The increasing size of the QD makes the volume larger, which in turn leads to weak confinement. DM elements as a function of the electric field and the height (radius) of the oblate (prolate) QD are studied, which improved understanding of the optical ACs, RICs, SHG and THG coefficients variation with the lateral and perpendicular electric field and size of the QD. The lateral electric field leads to a blueshift in ACs and RICs for the oblate QD and a redshift in prolate QDs; however, in oblate QD, while for the perpendicular field, blueshift is observed for both oblate and prolate QD for ACs and RICs. The WL thickness is a crucial tool for the behavior of nonlinear optical response. The spectra of resonance peaks are crucially changed with WL and the electric field. The WL layer effect enhances the difference of oblate and prolate QD, as with an increase in WL, the blueshift is obtained for oblate QD with increasing electric field, and no such shift is observed in prolate QD. The lateral electric field increases the amplitude of SHG and THG for oblate QD but it decreases the amplitude of prolate QD. The perpendicular electric field decreases the amplitude of SHG and increases the amplitude of THG with blueshift in both. WL thickness causes redshift in SHG and THG resonance peaks. Hence, the electric field, not just the shape and size of the QD, can be a regulating parameter. We can control the optical properties of the nanostructures by adjusting the electric field strength and the wetting layer instead of the size, which is an expensive operation. We also investigated that the bandgap is tunable with QD size and the electric field. As SiGe is generally utilized in electronic parts and optoelectronic gadgets in this way, with the information on the variety of the bandgap and nonlinear optical properties with the electric field, we can revolutionalize the planning of the optoelectronic gadgets by keeping the WL and the electric field relationship with the properties.
